# LC-DFSA: Low Complexity Dynamic Frame Slotted Aloha Anti-Collision Algorithm for RFID System

**DOI:** 10.3390/s20010228

**Published:** 2019-12-31

**Authors:** Zhaozhe Jiang, Bo Li, Mao Yang, Zhongjiang Yan

**Affiliations:** School of Electronics and Information, Northwestern Polytechnical University, Xi’an 710072, China

**Keywords:** radio frequency identification (RFID), dynamic frame slotted Aloha (DFSA), anti-collision, low complexity

## Abstract

With the rapid development of the Internet of Things (IoT), the radio frequency identification (RFID) system becomes increasingly important. Tag identification is a basic problem of the RFID system, whose purpose is to inventory tags. However, in recent years, it requires a very short time for massive tag identification, which brings serious challenges. The traditional Aloha based anti-collision algorithms have disadvantages of either low efficiency or high complexity. Therefore, this article proposes a low complexity dynamic frame slotted Aloha (DFSA) anti-collision algorithm, named LC-DFSA. The reader can estimate the range of tag numbers according to the last frame size, the number of successful slots and the ratio of idle slots. Then the optimal frame size can be calculated. Complexity analysis is deployed in this article, and we validate the correctness of the analysis. Through our simulations, LC-DFSA outperforms other schemes in both the average access efficiency and the algorithm complexity. It also can be conveniently applied to engineering implementations.

## 1. Introduction

Over the years, with the rapid development of information technology, the Internet of Things (IoT) has begun to penetrate into people’s lives [1]. IoT realizes the Internet of everything through sensor technology, communication technology, network technology and so on [2]. Radio frequency identification (RFID) is an important part of IoT.

Tag identification is a basic and important problem in the RFID system [3]. It is implemented by information exchanged between a reader and many tags, which are attached to different objects [4]. Nowadays, due to the higher demand of logistics [5], warehouse inventory [6], vehicle [7] and other scenarios [8,9,10], tag identification has to face to tricky challenges: to identify massive tags in a very short amount of time. Therefore, high efficient tag identification with low complexity is quite important.

As an important issue of tag identification, response collision needs attentions. In the ISO/IEC 18000 standard, solutions to collision problems are mentioned. Anti-collision techniques can be roughly divided into two types: protocols based on Aloha and protocols based on Binary Tree. For example, in ISO/IEC 18000-6 [11], mode A is a kind of Aloha based protocol, while mode B is a protocol based on Binary Tree. Mode A explicitly proposes the concept of the Dynamic Frame Slotted Aloha (DFSA) algorithm. However, it does not specify how to achieve it.

Some specific DFSA algorithms have been put forward. Mustapha et al. [12] proposed a Tag Estimation Method (TEM) that combined Bayesian and lower bound estimating. Chu et al. [13] estimated tag population through an enhanced Bayesian Method used in the physical layer. Wang et al. [14] used the secant iteration method to estimate the number of tags. Chen [15] performed once early judgment to adjust frame sizes. HajMirzaei [16] proposed a TEM based on Manchester encoding, and Chen [17] used the Maximum a Posteriori (MAP) to estimate the tag number. They are either with low efficiency or with high complexity considering the limited power in passive RFID systems.

In order to reduce the complexity of anti-collision algorithms in the RFID system while maintaining high efficiency, this paper proposes a low complexity anti-collision algorithm based on DFSA, named LC-DFSA. We calculate the demarcation points of different frame sizes through theoretical analyses, and we also analyze the border values of tag numbers in the case of no idle slots, no successful slots and full collision slots. Then the frame size can be kept in an optimizing value according to the demarcation points and border values. Moreover, the above values used in LC-DFSA can be stored in the memory of the reader in advance, which means that the LC-DFSA algorithm only performs some simple calculation and comparison. Thus, it lowers the complexity of the passive RFID system. The results of the analyses and simulations show that the complexity of LC-DFSA is lower than that of Chen [15] and HajMirzaei [16], and the access efficiency of the proposed LC-DFSA achieves 0.345 for the massive tags scenario, which outperforms Chen [15] and HajMirzaei [16].

The contributions of this paper are summarized as follows:

- This paper proposes a low complexity anti-collision algorithm named LC-DFSA, which can be conveniently applied to engineering implementations.
- Meanwhile, the computational and signaling complexity of LC-DFSA is low for a passive RFID system, and the compatibility for the standard framework is good as well.

The rest of this paper is arranged as follows. In the Section 2, Aloha based algorithms are briefly reviewed. The LC-DFSA algorithm will be introduced in the Section 3. After that, Section 4 concludes simulation results and analysis, and Section 5 summarizes this paper in the end.

## 2. Motivation

### 2.1. Brief Introduction to Aloha Based Anti-Collision Algorithm

Anti-collision algorithms based on Aloha can be roughly divided into five categories [18]: Pure Aloha (PA), Slotted Aloha (SA), Frame Slotted Aloha (FSA), DFSA and Enhanced Dynamic Frame Slotted Aloha (EDFSA).

Pure Aloha (PA): When tags are in the communication range of a reader, they will automatically reply their ID to the reader immediately. Then it will result in three situations: successful identification, complete collision and partial collision. If current identification is failed, the tag will respond again after a random interval of time, and the above steps repeat until all tags are identified by the reader successfully.

Slotted Aloha (SA): Continuous time is split into discrete slots. All tags can only respond at the beginning of the slot. Since the slot duration is longer than that of response, SA avoids partial collisions and improves the efficiency of RFID.

Frame Slotted Aloha (FSA): Several slots make up one frame. Each tag can only respond once in one frame, which can alleviate the problem of frequent response of some tags.

DFSA: After a frame, the reader can dynamically adjust the size of next frame according to the current situation. Therefore, it can improve the efficiency of the RFID system no matter how many tags there are.

Enhanced Dynamic Frame Slotted Aloha (EDFSA): Because the maximum frame size in mode A is 256 [11], when the tag population is larger, the efficiency of the RFID system decreases rapidly. Therefore, tags can be grouped and identified in turn when the tag amount is larger. EDFSA has greatly improved the efficiency of large-scale tag identification.

### 2.2. Motivation

**Figure 1:**
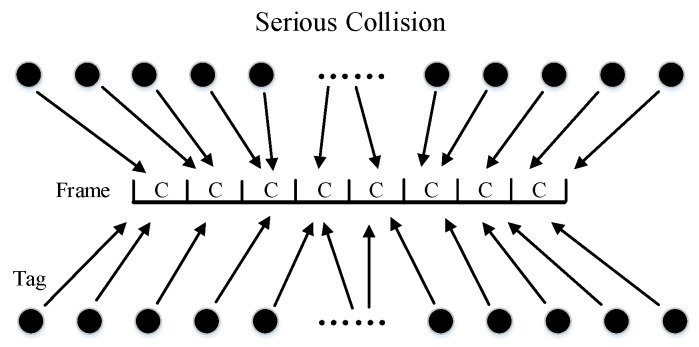
Large Amount of Tags.

**Figure 2:**
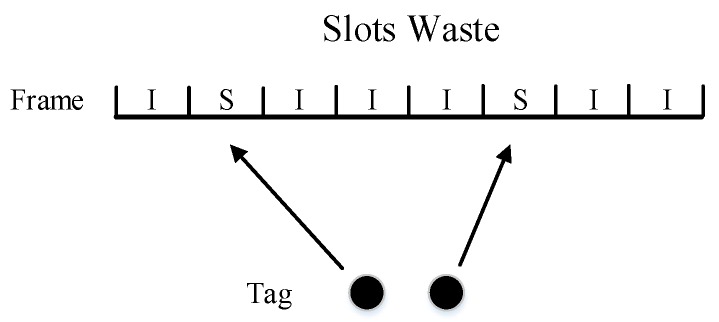
Small amount of tags.

It is always the case in tag identification that the tag population is unknown. As shown in Figure 1 and Figure 2, when the tag number is much larger than the frame size, collisions will be serious. It will spend more frames to identify all tags. But when the tag number is much smaller than the frame size, plenty of idle slots will be wasted. It will also lead to the reduction of overall efficiency. Therefore, we need an algorithm that can adjust the frame size dynamically. In this way, no matter what the tag number is, tag identification can maintain high efficiency all the time.

## 3. Proposed LC-DFSA Algorithm

### 3.1. Key Idea

**Figure 3:**
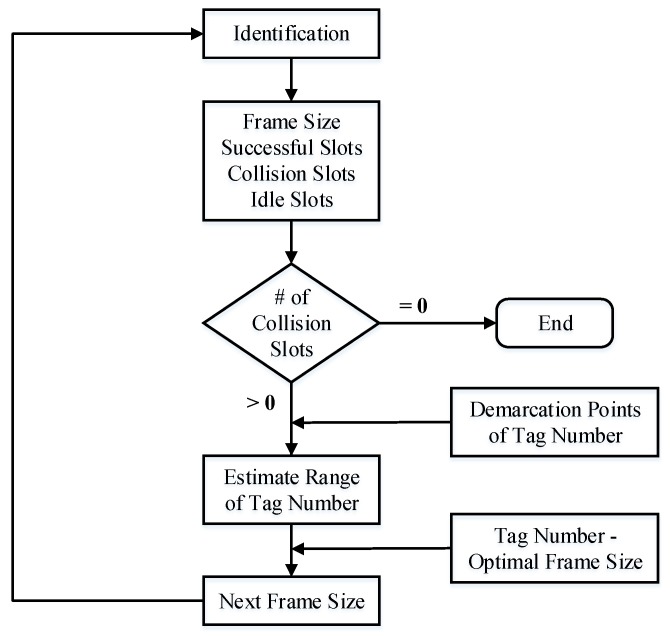
Tag identification process.

If the tag number is known, we can calculate the appropriate frame size to maximize access efficiency. Since frame sizes may not be continuous natural numbers, different tag numbers may correspond to the same optimal frame size. Therefore, even if the number of tags is unknown, if the approximate range of tag numbers can be estimated, the optimal frame size also can be determined. Figure 3 shows the process of tag identification.

In this paper, the corresponding relationship between tag numbers and optimal frame sizes is obtained through access efficiency. Then, the optimal size of next frame can be calculated by the last frame size, successful slots number and the ratio of idle slots.

### 3.2. Optimal Frame Size

Assuming that the tag number is fixed, it is obvious that the frame size can be neither too large nor too small. Therefore, in the case of a fixed tag number, it is necessary to determine the optimal frame size, so as to reach the highest efficiency. Table 1 shows some notations which will be used in the theoretical analysis.

For a particular slot, the probability that each tag chooses the slot is $\frac{1}{N}$ and the probability of selecting other slots is $1-\frac{1}{N}$. So, the probability of a successful slot is given by:

(1) $$P_{S}=\frac{1}{N}{\left(1-\frac{1}{N}\right)}^{M-1}.$$

and the probability that a tag is successful in a frame is:

(2) $$NP_{S}={\left(1-\frac{1}{N}\right)}^{M-1}.$$

Then we can get the expected successful tags number in a frame, which is:

(3) $$N_{S}=M{\left(1-\frac{1}{N}\right)}^{M-1}.$$

Therefore, the expected value of access efficiency can be calculated as:

(4) $$\eta_{S}=\frac{N_{S}}{N}=\frac{M}{N}{\left(1-\frac{1}{N}\right)}^{M-1}.$$

As we can see from Equation (4), $\eta_{S}$ is a function of M and N. When M is fixed, $\eta_{S}$ will be a unary function of N. In order to maximize $\eta_{S}$, perform:

(5) $$\frac{d\eta_{S}}{dN}=0.$$

Then we get:

(6) N=M.

**Figure 4:**
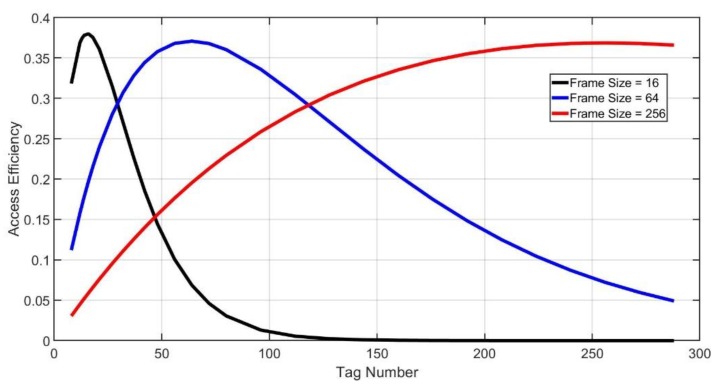
Access efficiency.

As shown in Figure 4, the maximum access efficiency will be realized if the frame size is equal to the tag number.

### 3.3. Tag Number—Optimal Frame Size Table

In ISO/IEC 18000-6, the frame size can only be taken from the several discrete values [11], assuming that frame sizes are the values from a discrete integer sequence $\left\{N_{1},N_{2},N_{3},\cdots \cdots ,N_{\max}\right\}$. However, the range of tag numbers belongs to positive integers. It is almost impossible that the frame size is exactly the same as the tag number all of the time. Therefore, when the tag number is fixed, we need to confirm the available optimal frame size to maximize access efficiency. Eventually, we can get the relationship between optimal frame sizes and tag numbers.

For a fixed tag number M, there are many frame sizes that can be chosen. Through $\eta_{S}$, we can calculate the maximum access efficiency and determine the optimal frame size.

As we can see from Equation (4), $\eta_{S}$ is a function of tag number M and frame size N, and the demarcation points of tag numbers between $N_{i}$ and $N_{i+1}$ can be calculated through the following equation:

(7) $$\eta_{S}(M,N_{i})=\eta_{S}(M,N_{i+1},).$$

The demarcation points $M_{i}^{Dem}$ are the solution of Equation (7):

(8) $$M_{i}^{Dem}=1+\frac{\mathrm{lg}\frac{N_{i+1}}{N_{i}}}{\mathrm{lg}\frac{N_{i}(N_{i+1}-1)}{N_{i+1}(N_{i}-1)}}.$$

Demarcation points are the boundary values of the ranges of tag numbers. If the actual tag number is between $M_{i}^{Dem}$ and $M_{i+1}^{Dem}$, then $N_{i+1}$ will become the optimal size of next frame. When frame sizes are restricted to the power of 2, the demarcation points are shown, as in Table 2.

The tag number—optimal frame size table is shown in Table 3. For example, if the tag number is 70, the RFID system will get the highest efficiency when the frame size is 64. Efficiency will be lower if the frame size is 32 or any other number.

### 3.4. LC-DFSA Algorithm

The size of the last frame, the successful slots number and the ratio of idle slots are used in LC-DFSA to estimate the range of the tag number and to calculate the optimal next frame size.

The probability of appearance of an idle slot is given by:

(9) $$P_{I}={\left(1-\frac{1}{N}\right)}^{M}.$$

Then the expected idle slots number in a frame is:

(10) $$NP_{I}=N{\left(1-\frac{1}{N}\right)}^{M}.$$

Therefore, the expected ratio of idle slots can be calculated as:

(11) $$\eta_{I}=\frac{NP_{I}}{N}={\left(1-\frac{1}{N}\right)}^{M}.$$

**Figure 5:**
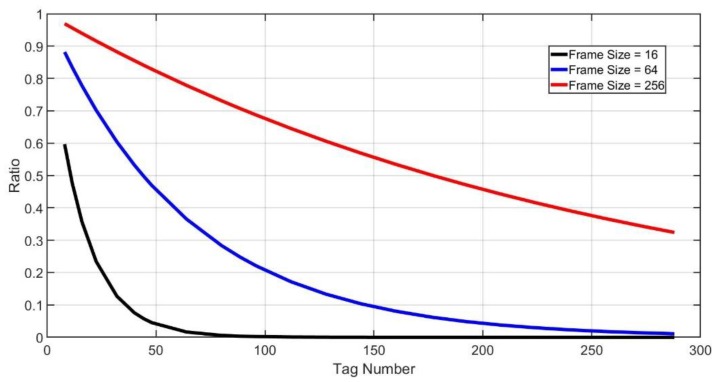
Idle slot ratio.

Figure 5 shows the relationship between tag numbers and idle slot ratios when frame sizes are fixed. Idle slot ratio $\eta_{I}$ is a monotonic minus function of tag number M. Therefore, we can estimate the range of tag numbers by $\eta_{I}$ due to its monotonicity. Through Equation (8), we can calculate tag number demarcation points, which are $M_{i}^{Dem}\;(i=1,\;2,\;3,\;4,\;\cdots \cdots ,\max-1)$. In order to determine the optimal size of next frame, the demarcation points that we actually use are:

$$M_{i}^{Dem}+N_{S}^{\prime }\;(i=1,\;2,\;3,\;4,\;\cdots \cdots ,\max-1).$$

$N_{S}^{\prime }$ is the actual successful slot number of the last frame. According to the size of the last frame, the reader can get $\eta_{I}$ for each demarcation point. Then the reader can calculate the actual idle slot ratio $\eta_{I}^{\prime }=\frac{N_{I}^{\prime }}{N}$. Therefore, the range of current tag number $M_{Est}$ can be estimated by comparing $\eta_{I}^{\prime }$ and $\eta_{I}$ of the demarcation points.

**Figure 6:**
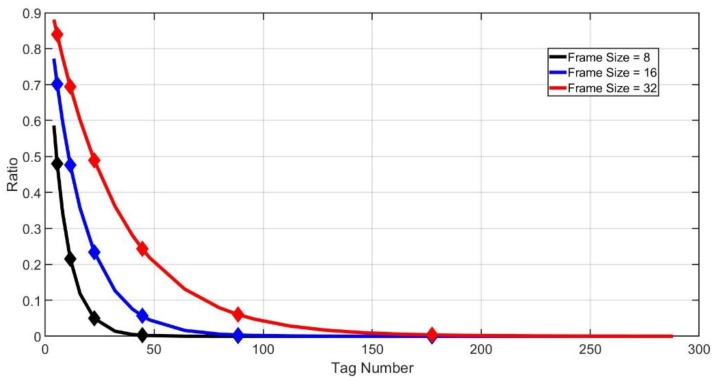
Demarcation points ($N_{S}^{\prime }=0$).

For instance, assume that the last frame size is 32 and $\eta_{I}^{\prime }$ is between $\eta_{I}(M_{3}^{Dem}+N_{S}^{\prime })$ and $\eta_{I}(M_{4}^{Dem}+N_{S}^{\prime })$. $M_{Est}$ will be estimated as $\left(M_{3}^{Dem}+N_{S}^{\prime },\;M_{4}^{Dem}+N_{S}^{\prime }\right)$. Then, the size of next frame will be $N_{4}$, which is 16. Figure 6 shows the demarcation points when the last frame size is small, assuming $N_{S}^{\prime }=0$.

As we can see from Figure 6, when the frame size is much smaller than the tag number, the idle slot ratio curve descends rapidly. It may not be possible to accurately determine the range of tag numbers. For example, assume that the size of the last frame is 8 and $N_{S}^{\prime }=0$. When $N_{i}^{\prime }=0$, the next frame size will be $N_{\max}$ according to our algorithm above. However, the actual tag number may be only 50. It will waste plenty of slots in this case. To solve the problem, we can analyze the border of successful, idle and collision slot ratios.

**Figure 7:**
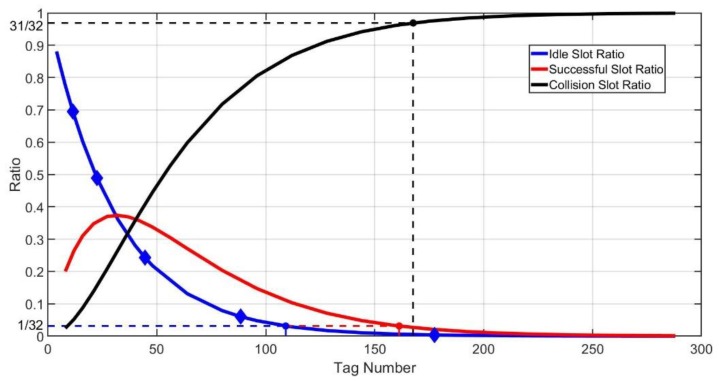
Border of idle, successful and collision slot ratio.

Figure 7 shows the ratios of idle, successful and collision slots when the frame size is 32. If the tag number is larger than $M_{Idle}^{Border}$, the probability of $N_{I}^{\prime }=0$ will be very high. $M_{Idle}^{Border}$ is the solution of Equation (12).

(12) $${\left(1-\frac{1}{N}\right)}^{M}=\frac{1}{N}.$$

If the tag number is larger than $M_{Suc}^{Border}$, the actual number of successful slots will be likely to reduce to 0. $M_{Suc}^{Border}$ is the solution of Equation (13).

(13) $$\frac{M}{N}{\left(1-\frac{1}{N}\right)}^{M-1}=\frac{1}{N}.$$

In the case of that the tag number is larger than $M_{Coll}^{Border}$, all slots in the frame will be almost in collision. $M_{Coll}^{Border}$ is the solution of Equation (14).

(14) $${\left(1-\frac{1}{N}\right)}^{M}+\frac{M}{N}{\left(1-\frac{1}{N}\right)}^{M-1}=\frac{1}{N}.$$

Therefore, as Algorithm 1 presents, when $N_{I}^{\prime }=0$ and $N_{S}^{\prime }>0$, it means that the actual remaining tags may not be so many. The tag number will be estimated as $M_{Idle}^{Border}$ conservatively, and the next frame size can be calculated through the relationship between tag numbers and optimal frame sizes. Meanwhile, when $N_{I}^{\prime }=0$ and $N_{S}^{\prime }=0$, the next frame size will be determined through $M_{Coll}^{Border}$, and if $N_{I}^{\prime }>0$, we can calculate the next frame size according to $M_{Est}$ directly. Then, start a new frame and repeat above steps until all the tags are identified.
**Algorithm 1** LC-DFSA1: After a frame, get N, $N_{I}^{\prime }$ and $N_{S}^{\prime }$.
2: **If**
$N_{I}^{\prime }+N_{S}^{\prime }<N$
3:  **If**
$N_{I}^{\prime }=0$
4:   **If**
$N_{S}^{\prime }=0$
5:    $N_{Next}$ will be calculated by $M_{Coll}^{Border}$.
6:   **Else**
7:    $N_{Next}$ will be calculated by $M_{Idle}^{Border}$.
8:  **Else**
9:   Calculate $\eta_{I}^{\prime }$ and $\eta_{I}(M_{i}^{Dem}+N_{S}^{\prime })$.
10:  Compare $\eta_{I}^{\prime }$ and $\eta_{I}(M_{i}^{Dem}+N_{S}^{\prime })$. And get $M_{Est}$.
11:  $N_{Next}$ will be calculated by $M_{Est}$ directly.
12:  Start a new frame with $N_{Next}$ slots.
13: **Else**
14:  Tag inventory completes.

### 3.5. Complexity Analysis

As shown in Table 4, the LC-DFSA algorithm performs normal signaling interactions of standard ISO/IEC 18000-6, which leads to its lowest signaling complexity. Chen [15] could achieve low complexity based on the normal signaling interactions of the Q-algorithm. The signaling complexity of Wang and Chen [17] would be a little bit higher due to their continuous frame sizes. However, HajMirzaei increased the signaling overhead a lot to improve the estimation accuracy of tag numbers. Therefore, the signaling complexity of HajMirzaei was the highest.

As for computational complexity, all the demarcation points and border values of LC-DFSA can save in the memory of the reader. Thus, the computational complexity of the LC-DFSA algorithm is low. Chen [15] estimated the tag number as $S_{i}+2.39C_{i}$, which needs few computing resources. Chen [17] would calculate the probability of each possible number of tags through the multinomial distribution formula, and then would find the tag number, which has the maximum probability. It costs a lot of resources, so it is unavailable for engineering implementation. Wang solved a transcendental equation by secant iteration and eliminated the pseudo solution, which increased its computational complexity; HajMirzaei only performed some simple calculations and comparisons, which led to its low computational complexity.

Standard compatibility is also important for anti-collision algorithms. Chen [15] could only achieve within the framework of the UHF Class-1 Generation-2 RFID Standard [19], which lowered the compatibility for standards. However, the others can be applied in not only ISO/IEC 18000-6A, but also ISO/IEC 18000-6C, whose framework is the same as UHF Class-1 Generation-2. They have better standard compatibility than Chen [15].

## 4. Simulation and Analysis

MATLAB 2018 is used for our simulations. The number of tags ranges from 1000 to 10,000. All simulation results are the average value of 5000 simulations. The initial frame sizes of all the algorithms are set as 1024 in our simulations. The frame sizes $\left\{N_{1},N_{2},N_{3},\cdots \cdots ,N_{\max}\right\}$ in the LC-DFSA algorithm are restricted to the power of 2, and the check point of Chen [15] is $\left(\frac{3L}{4}\right)\mathrm{th}$.

**Figure 8:**
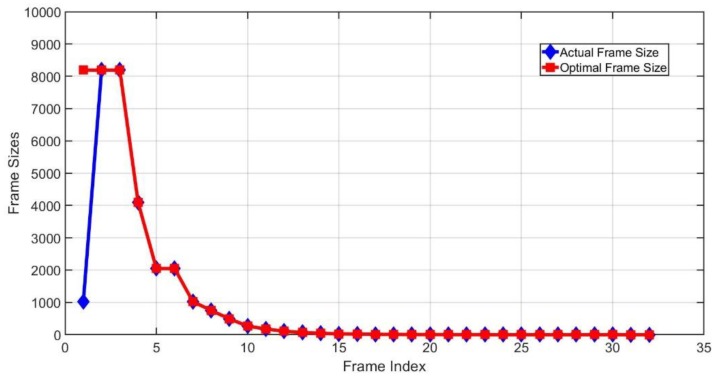
Actual and optimal frame size.

In Figure 8, the blue line represents the actual frame sizes of the LC-DFSA algorithm, while the red line shows the optimal frame sizes. The tag number is 10,000 and the initial frame size is set as 1024. In this case, 10,000 tags can be identified after 30 frames. According to the simulation results, after the second frame, the actual frame sizes are consistent with the optimal frame sizes, which shows the effectiveness of the LC-DFSA algorithm.

**Figure 9:**
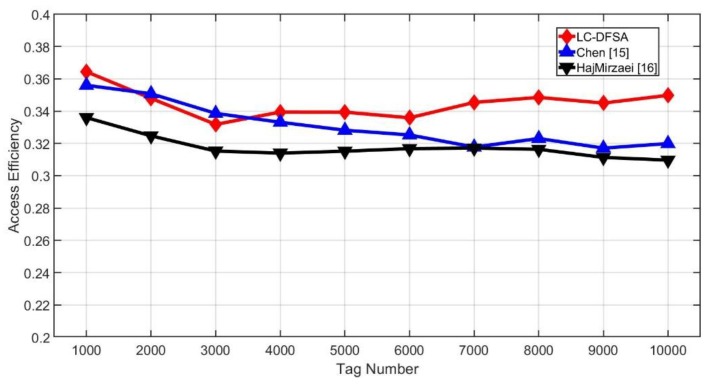
Access efficiency of different algorithms.

Figure 9 shows the access efficiency of different algorithms, which is also known as throughput. From the results, HajMirzaei has the worst performance among these three algorithms. When the tag number is smaller than 4000, the difference between Chen’s efficiency and LC-DFSA’s efficiency is small, and as the tag number increases further, the efficiency of Chen decreases. The accuracy of $S_{i}+2.39C_{i}$ is worse when the tag number and the frame size are mismatched with each other. The average efficiency of Chen is 0.3309 and that of HajMirzaei is 0.3176. The proposed LC-DFSA algorithm is 0.3448, which is the highest. In this way, the LC-DFSA algorithm improves 4.2% when compared with Chen, and it is an improvement of 5.4% when compared with HajMirzaei.

**Figure 10:**
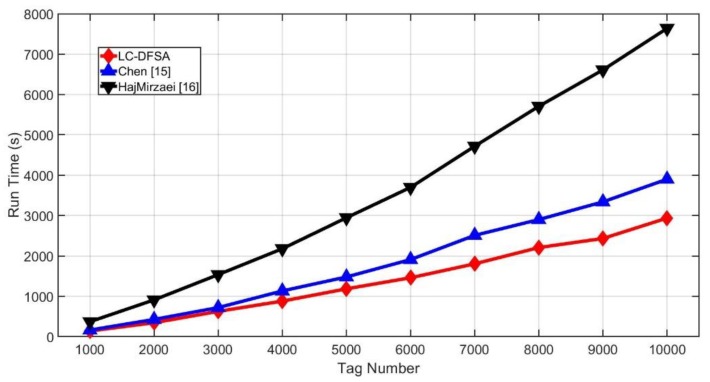
Simulation run time of different algorithms.

From the view of algorithm complexity, Chen, LC-DFSA and HajMirzaei only perform some fixed calculation and comparison, so the complexity of them are all O(1). Besides, Figure 10 shows the simulation run time of each algorithm. To some extent, it can be proved that the LC-DFSA algorithm has the lowest complexity. Collision analysis by Manchester encoding increases the overhead of HajMirzaei, and Chen performs the examination in the middle of the frame, which will lead to more frames. Moreover, frame sizes of LC-DFSA are discrete while frame sizes of HajMirzaei can be any natural number. It also lowers the complexity of LC-DFSA.

**Figure 11:**
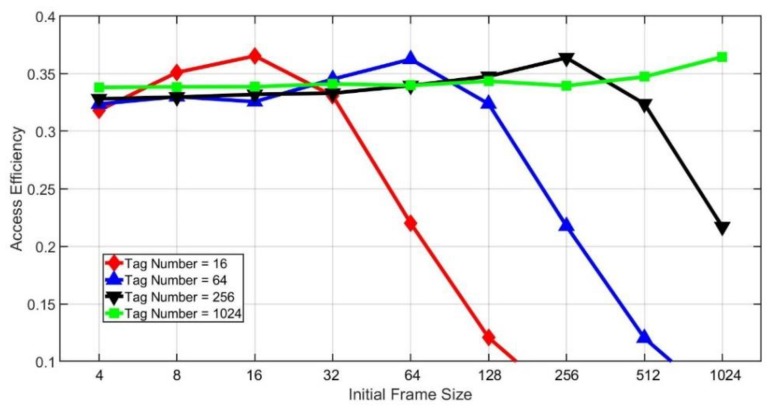
Access efficiency of different tag numbers.

**Figure 12:**
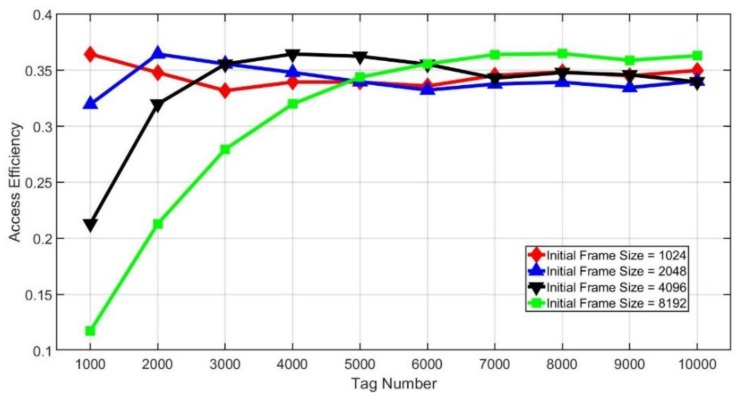
Access efficiency of different initial frame sizes.

Figure 11 and Figure 12 show the effect of different initial frame sizes on the access efficiency. As we can see from Figure 11, the initial frame size has a great impact on efficiency. However, if the tag number is larger, then the influence of the initial frame size will be smaller. From Figure 12, it is found that when the initial frame size is small compared with the tag number, there will be stable access efficiency no matter how large the tag amount is. However, when the initial frame size is larger, the efficiency drops dramatically if the tag number is small. Besides, we can also find that there will be the maximum efficiency when the initial frame size is the same as the number of tags.

## 5. Conclusions and Future Work

This article proposes the LC-DFSA algorithm with the purpose of complexity reduction and efficiency improvement for RFID systems. The reader can estimate the range of tag numbers according to the demarcation points and border values, which can be calculated through theoretical analyses. Then the size of next frame can be determined by the relationship between tag numbers and optimal frame sizes. Through our simulations, it is found that the complexity of the LC-DFSA algorithm is lower than that of Chen and HajMirzaei, and LC-DFSA also improves efficiency when compared with Chen and HajMirzaei. Therefore, the proposed LC-DFSA algorithm has the best comprehensive performance.

We still have some work to do in the future. For DFSA algorithms, the initial frame size has an effect on access efficiency. We will try to analyze different initial frame sizes separately so that the frame size can approach the optimal frame size as quickly as possible.

## Figures and Tables

**Table 1:** Notations.

Notation	Description
M	Tag number
N	Frame size
$N_{S}$	Expected number of successful tags in a frame
$P_{S}$	Probability that a tag is successful in a slot
$P_{I}$	Probability that a slot is idle
$\eta_{S}$	Expected ratio of successful slots in a frame
$\eta_{I}$	Expected ratio of idle slots in a frame

**Table 2:** Values of demarcation points.

**Demarcation Points**	$M_{2}^{Dem}$	$M_{3}^{Dem}$	$M_{4}^{Dem}$	$M_{5}^{Dem}$	$M_{6}^{Dem}$	…
**Values**	5.4966	11.0466	22.1391	44.3208	88.6827	…

**Table 3:** Tag number—optimal frame size.

Numbers of Tags	Optimal Frame Sizes
3~5	4
6~11	8
12~22	16
23~44	32
45~88	64
89~177	128
178~355	256
…	…

**Table 4:** Complexity comparison.

Different Algorithms	Signaling Complexity	Computational Complexity	Standard Compatibility
Wang [14]	Normal	Normal	Good
Chen [15]	Low	Low	Normal
HajMirzaei [16]	High	Low	Good
Chen [17]	Normal	High	Good
LC-DFSA	Low	Low	Good

## References

[1] Jayadi R., Lai Y.C., Lin C.C. (2017). Efficient Time-Oriented Anti-Collision Protocol for RFID Tag Identification. Comput. Commun..

[2] Su J., Sheng Z., Leung V.C., Chen Y. (2019). Energy Efficient Tag Identification Algorithms for RFID: Survey, Motivation and New Design. IEEE Wirel. Commun..

[3] Khalil G., Doss R., Chowdhury M. (2019). A Comparison Survey Study on RFID Based Anti-Counterfeiting Systems. J. Sens. Actuator Netw..

[4] Mbacke A.A., Mitton N., Rivano H. (2018). A Survey of RFID Readers Anticollision Protocols. IEEE J. Radio Freq. Identif..

[5] Zhou W., Jiang N., Yan C. (2019). Research on Anti-Collision Algorithm of RFID Tags in Logistics System. Procedia Comput. Sci..

[6] Biswal A.K., Jenamani M., Kumar S.K. (2018). Warehouse efficiency improvement using RFID in a humanitarian supply chain: Implications for Indian food security system. Transp. Res. Part E Logist. Transp. Rev..

[7] Ravi S., David A., Imaduddin M. (2018). Controlling & Calibrating Vehicle-Related Issues Using RFID Technology. SSRN Electron. J..

[8] Amato F., Torun H.M., Durgin G.D. (2018). RFID Backscattering in Long-Range Scenarios. IEEE Trans. Wirel. Commun..

[9] Parada R., Melià-Seguí J., Pous R. (2018). Anomaly Detection Using RFID-Based Information Management in an IoT Context. J. Organ. End User Comput..

[10] Yan L., Xiong D. (2018). Mobile motion robot indoor passive RFID location research. Int. J. RF Technol. Res. Appl..

[11] ISO/IEC CD 18000-6 (2004). Information Technology—Radio Frequency Identification (RFID) for Item management—Part 6: Parameters for Air Interface Communications at 860–930 MHz. http://www.youwokeji.com.cn/down/18000-6.pdf.

[12] Benssalah M., Djeddou M., Dahou B., Drouiche K., Maali A. (2018). A cooperative Bayesian and lower bound estimation in dynamic framed slotted ALOHA algorithm for RFID systems. Int. J. Commun. Syst..

[13] Chu C., Wen G., Huang Z., Su J., Han Y. (2019). Improved Bayesian Method with Collision Recovery for RFID Anti-collision. Proceedings of the International Conference on Artificial Intelligence and Security.

[14] Wang Z., Huang S., Fan L., Zhang T., Wang L., Wang Y. (2018). Adaptive and dynamic RFID tag anti-collision based on secant iteration. PLoS ONE.

[15] Chen W. (2014). A Fast Anticollision Algorithm for the EPCglobal UHF Class-1 Generation-2 RFID Standard. IEEE Commun. Lett..

[16] HajMirzaei M. (2019). Novel tag estimation method by use of Manchester coding in RFID systems. Int. J. Commun. Syst..

[17] Chen W.T. (2009). An Accurate Tag Estimate Method for Improving the Performance of an RFID Anticollision Algorithm Based on Dynamic Frame Length ALOHA. IEEE Trans. Autom. Sci. Eng..

[18] Klair D.K., Chin K.W., Raad R. (2010). A Survey and Tutorial of RFID Anti-Collision Protocols. IEEE Commun. Surv. Tutor..

[19] EPCglobal (2008). EPC Radio-Frequency Identity Protocols Class-1 Generation-2 UHF RFID Protocol for Communications at 860 MHz–960 MHz.

